# Dashboard Intervention for Tracking Digital Social Media Activity in the Clinical Care of Individuals With Mood and Anxiety Disorders: Randomized Trial

**DOI:** 10.2196/74212

**Published:** 2025-11-11

**Authors:** Leslie Miller, Tenzin C Lhaksampa, Alex Walker, Carlos Aguirre, Matthew DeCamp, Keith Harrigian, Jennifer Meuchel, Aja M Meyer, Brittany Nesbitt, Sazal Sthapit, Jason Straub, Danielle Virgadamo, Ayah Zirikly, Mark Dredze, Margaret S Chisolm, Peter P Zandi

**Affiliations:** 1 Department of Psychiatry and Behavioral Sciences, School of Medicine, Johns Hopkins University, 550 North Broadway, Suite 201, Baltimore, MD, 21218, United States, 1 4106141923; 2 Department of Mental Health, Bloomberg School of Public Health, Johns Hopkins University, Baltimore, MD, United States; 3 Department of Computer Science, Whiting School of Engineering, Johns Hopkins University, Baltimore, MD, United States; 4 Department of Internal Medicine, University of Colorado Anschutz Medical Campus, Aurora, CO, United States; 5 Center for Behavioral Health, Johns Hopkins All Children's Hospital, St. Petersburgh, FL, United States; 6 Center for Developmental Behavioral Health, Kennedy Krieger Institute, Baltimore, MD, United States; 7 Department of Computer Science, School of Engineering and Applied Science, George Washington University, Washington, DC, United States

**Keywords:** social media, mood disorders, anxiety, measurement-based care, randomized trial, electronic dashboard

## Abstract

**Background:**

Digital social activity, defined as interactions on social media and electronic communication platforms, has become increasingly important. Social factors impact mental health and can contribute to depression and anxiety. Therefore, incorporating digital social activity into routine mental health care has the potential to improve outcomes.

**Objective:**

This study aimed to compare treatment augmented with an electronic dashboard of patient’s digital social activity versus treatment-as-usual on patient-rated outcomes symptoms of depression in a randomized trial of patients with mood and anxiety disorders.

**Methods:**

We developed a personalized electronic dashboard summarizing a participant’s digital social activity. This dashboard, collaboratively discussed during mental health visits, was used to augment clinical care and tested in a randomized trial against treatment-as-usual. Clinicians and patients were recruited from outpatient psychiatry clinics. Patients were eligible if they were 12 years or older and were receiving treatment for a mood or anxiety disorder. Psychiatric symptoms measures for depression (primary outcome measure) and anxiety (secondary outcome measure) were obtained at each clinic visit as part of measurement-based standard of care. Baseline and 3-month follow-up assessments included a measure of mental health status and therapeutic alliance measure. Collateral information and clinical action scale were also collected at each visit.

**Results:**

A total of 103 patients consented to participate, 97 of whom were randomized to the dashboard arm (n=49) or the treatment-as-usual arm (n=48). There were no differences in psychiatry symptom rating scores or mental health status between the two arms. However, there was a significant increase in the discussion of digital social activity with the intervention, and it did not appear to change patient therapeutic alliance.

**Conclusions:**

The incorporation of a personalized electronic dashboard into clinical care was feasible and led to an increased discussion of digital social activity, but there was no impact on mental health outcomes.

## Introduction

This study reports on the results of a randomized trial for patients with mood or anxiety disorders comparing treatment-as-usual (TAU) augmented with an electronic dashboard provided to clinicians for tracking their patients’ digital social activity versus TAU. Digital social activity is defined as patient interactions on social media or through electronic communication such as texting. It is well established that social factors—such as social support, stressful life events, social isolation, discrimination, socioeconomic status, family dynamics, peer networks—are important in the development and progression of depression and anxiety disorders [1-5]. Moreover, our social lives, especially among adolescents and young adults, are increasingly carried out online via various digital platforms [6]. Therefore, providing a clinical tool allowing clinicians and patients to collaboratively monitor their digital social activity could help identity issues to be addressed in treatment and improve clinical outcomes [7].

A growing body of research suggests that useful clinical insights about patients’ mental health can be gleaned from their digital social activity. Both clinicians and patients have reported they feel comfortable discussing the patients’ social media activity in therapy sessions [8-10]. Moreover, several studies [8,11,12] have found that many clinicians will ask patients about their electronic communication on social media because they believe this information is helpful in providing more effective treatment [12]. Additionally, a number of studies have shown that quantifiable signals derived from patterns of activity and language used on different social media and electronic communication platforms can be used to develop computational models that help distinguish the presence of different mental illnesses [13-22], as well as predict meaningful changes in the clinical trajectories of these illnesses [14,23-26]. More recently, two additional studies were conducted. In the first, an electronic dashboard was developed to display the results of computational analyses carried out with data on patients’ digital social activity, but it was not formally tested in clinical practice [27,28]. In the second, patient digital activity was tracked as part of clinical care; although it was feasible, it did not improve mental health outcomes [29]. Taken together, these studies provide a compelling rationale for further study on the potential benefits of systematically monitoring patient digital social activity to assist in clinical care.

Towards this end, we developed an electronic dashboard for displaying information about patients’ digital social activity for clinicians to use with patients during routine clinical visits to inform treatment decisions. We envisioned the dashboard to be part of a next-generation measurement-based care program in which multiple sources of collateral information about patients, including patient-rated outcome measures, are available to inform collaborative treatment decisions between clinicians and patients. Consistent with the principles of a measurement-based care program [7,30], we hypothesized that providing the electronic dashboard could improve downstream patient outcomes through two mediating pathways. First, it could lead to earlier detection of issues in the patients’ social lives that would benefit from more timely intervention. Second, it may improve the therapeutic alliance by fostering more informed and open communication between patients and clinicians [31]. With this in mind, we set out to clinically test whether augmenting TAU with the electronic dashboard could improve treatment outcomes on clinical symptoms compared to TAU alone, and whether it did so by impacting either of the hypothesized mediating pathways.

## Methods

### Dashboard Intervention

The design and development of the electronic dashboard intervention have been described previously [7]. The personalized electronic dashboard was designed to display salient information about the patient’s digital social activity in easy-to-interpret graphical formats. This included displays of usage statistics such as the frequency of messages sent/received over the past week, the list of platforms used to send/receive the messages, and the timing of when the messages were sent/received during the day/week; content metrics describing the major thematic elements of the messages sent/received as characterized by, for example, linguistic inquiry and word count (LIWC) analysis and trends in the use of certain pronoun forms that may be indicative of mental health status [32-34]; and specific content including snippets of messages that were algorithmically selected to be representative of the major themes identified over the past week. Regarding the latter, the specific content was masked on the dashboard, and clinicians were instructed to unmask and review the content only after patient permission during clinical encounters.

The patient’s social media and electronic communications activity data used to generate the dashboard were collected using Bark [35]. Bark is a commercially available parental/caregiver monitoring app that scans children’s on-line activity, including over 30 social apps, web browsers, emails, and texts. We worked with the company to adapt the app to shut down alert features to use it exclusively for data collection. Bark had access only to anonymous study IDs. The collected data were transferred from Bark through an encrypted HTTPS/TLS connection to a Johns Hopkins University network drive. Data were then processed to remove images, videos, geolocation tags, and internet usage (not including search engine queries), leaving natural text that was further processed to mask personal identifiers by replacing them with synthetic alternatives [22]. Computational algorithms were run on the processed data to derive the measures and visual displays described. The personalized dashboard was securely delivered as an HTML to clinicians for review before each clinic visit. Clinicians had access only to their patients’ dashboards.

### Study Sample

The study was conducted at the Johns Hopkins Medical Institution and the Kennedy Krieger Institute, which is an independent hospital affiliated with the Johns Hopkins Medical Institution. We engaged collaborative care outpatient psychiatry clinics, in which patients see a psychiatrist for medication-assisted care and a master’s level therapist or doctoral level psychologist for psychotherapy, as study sites. We first recruited clinicians from these sites to participate in the study who then referred their potentially eligible patients to learn more about the study. Patients aged 12 years and above with a mood or anxiety disorder in care at any stage of their illness were eligible to participate in the trial. There were no exclusion criteria other than if the treating clinician determined that the patient was not able to provide informed consent.

### Study Procedures

This pragmatic trial was implemented in as close to a real-world setting as possible to facilitate participation and enhance the generalizability of the findings. Patients were randomized in a 1:1 ratio with blocks of size 2 within treating clinicians to either the intervention (the dashboard arm) or TAU (the TAU arm). Randomization was implemented using REDCap and an allocation table generated to determine the randomization sequence for assigning patients to the comparison arms in a process that was blinded to the clinicians and patients. This helped promote balance in the number of patients in the two treatment arms seen by each clinician to minimize the influence of differences between treating clinicians. For patients in the TAU arm, the treating clinician did not receive a clinical dashboard. For patients in the dashboard arm, clinicians had access to the electronic dashboard prior to every scheduled clinic visit. The clinician and patient collaboratively decided on the use of the dashboard in clinical sessions. After the initial visit to download the Bark app, there were no further study-specific visits, and patients were seen by their clinicians as clinically indicated. We considered the first regularly scheduled clinic visit with their clinician after the patient consented and downloaded the Bark app to be the study baseline visit. We originally planned to follow-up patients for 1 year, but due to the impact of the COVID-19 pandemic on the start of the trial and patient recruitment, we modified the protocol to follow-up patients for 3 months.

### Study Measures

The full set of measures collected during the trial and the schedule of their collection have been described previously [7]. Here, we describe the measures used to evaluate the trial results. Patients completed the 9-item Patient Health Questionnaire-9 (PHQ-9) [36] to measure depressive symptoms and the 7-item General Anxiety Disorder-7 (GAD-7) [37] to measure general anxiety symptoms as part of an established measurement-based care program program in which these measures are collected as standard of care through the electronic health record before each clinic visit. In addition, they completed the 13-item Short-Form 36 Health Survey Mental Health Component (SF36-MHC) at baseline and after 3 months [38] to measure overall mental health status, and the 12-item Working Alliance Inventory–Short Revised (WAI-SR) to measure therapeutic alliance on aspects related to the goals, tasks, and bonds in treatment. The clinicians completed the McLean Collateral Information and Clinical Actionability Scale (M-CICAS) [39] after every clinic visit with patients in the trial. The M-CICAS was developed to quantify the types of collateral information that are reviewed during mental health related clinical sessions and to understand how this collateral information translates into clinical decision-making. It is a 7-item scale divided into three parts that collect information on the collateral sources of information reviewed, clinical actions taken, and shared decisions made between a clinician and a patient in a clinical session.

Patient diagnoses were obtained from the electronic health records and defined based on the most recent International Classification of Diseases-10 codes assigned by treating clinicians at clinic visits. Diagnoses were categorized as any bipolar disorder, any depressive disorder, or any anxiety disorder. Patients assigned a diagnosis in more than one category were categorized in a hierarchical fashion in the order of priority of bipolar disorder, depression, then anxiety disorder.

### Statistical Analysis

 We provided basic descriptive statistics of the sample with comparisons between the two treatment arms made using *χ*^2^ tests. We then compared patient outcomes between the two treatment arms. The primary outcome was the difference in depressive symptoms as measured using the total PHQ-9 scores [36] over follow-up. Secondary outcomes included differences in overall mental health status as measured using the SF36-MHC scores [38] and general anxiety as measured using the total GAD-7 scores [37]. We first compared these outcomes by taking the means of the scores within patients over the follow-up and comparing the means between the two treatment arms using *t* tests. We then used random effects linear regression models to test for differences in repeated measures of the scores over follow-up. We included fixed effects terms for the treatment arm (dashboard versus TAU arms), as well as for time of follow-up in days and other potential confounders including age, sex, race, and diagnosis. We included random effects for individuals to account for the correlation in outcome measures within repeated measures of the same individual. The fixed effect term for treatment provided an estimate of the mean differences in the outcome measures between the two treatment arms controlling for time and the other covariates. Given that patients were pragmatically enrolled into the study regardless of their current mental health status as long as they were in care, we considered the results from this model to be our primary test of interest. However, we also compared changes in these outcomes scores over follow-up between the two treatment arms using *t* tests of the delta in scores from baseline to the last available measure and a random effects model similar to the one described above but this time including an interaction term between the treatment arm and time while controlling for the other patient covariates. We carried out a series of diagnostic tests to confirm the validity of the assumptions for using random-effects models with these data. Visual inspection of residual plots revealed evidence of heteroscedasticity, so we used robust SEs in all our final models.

In secondary analyses, we examined whether there were differences in the two treatment arms on measures of therapeutic alliance and the detection of clinically actionable targets, both of which we hypothesized mediate the therapeutic effect of the intervention. In particular, we tested whether there were differences between the two treatment arms on scores on the WAI-SR (which measured therapeutic alliance) and the M-CICAS (which captured information about the detection of clinically actionable targets and if subsequent treatment adjustments were made). For the WAI-SR score, we took the means of all measures over follow-up per patient and compared them using *t* tests as well as using linear regression controlling for length of follow-up and patient covariates. For the M-CICAS, which is captured after every clinic visit, we tallied at how many clinical encounters issues related to the patient’s social media activity or electronic communications were discussed and subsequent treatment adjustments were made. We then compared the number of times digital social activity was discussed using *t* tests as well as linear regression controlling for length of follow-up and patient covariates. In other secondary analyses, we carried out stratified analyses to test whether there were differences in the primary outcomes by patient age and by a measure of clinician adherence to using the dashboard intervention. We repeated all univariate tests described above using non-parametric Mann-Whitney *U* tests to address concerns about potential violations of the assumptions of *t* tests, such as normality or equal variances of the underlying data between groups, and we observed nearly identical results (results not shown).

We planned, at the beginning of the study, to recruit a sample size of 100 based on the calculation that such a sample would have 80% power to detect differences in the primary outcome between the two treatment arms with a Cohen *d* effect size of at least 0.5 assuming at least 3 repeated measures. We used the conventional threshold of *P*<.05 to declare findings statistically significant, with the understanding that the multiple tests of the secondary outcomes and analyses were exploratory in nature.

### Ethical Considerations

The study protocol was approved by the Johns Hopkins University School of Medicine Institutional Review Board (IRB #00184638). We obtained informed consent from both patients and clinicians who participated in the study. We obtained assent from patients who were aged <18 years and consent from their guardians. Patients were compensated for participation in the study as described previously [7]. All data were deidentified before data analyses were conducted.

## Results

The trial was initiated in April 2019, but due to delays in the start-up period that persisted through the onset of COVID-19 pandemic, the first patient was not randomized until October 2020. Enrollment was completed in February 2024.

**Figure 1.**
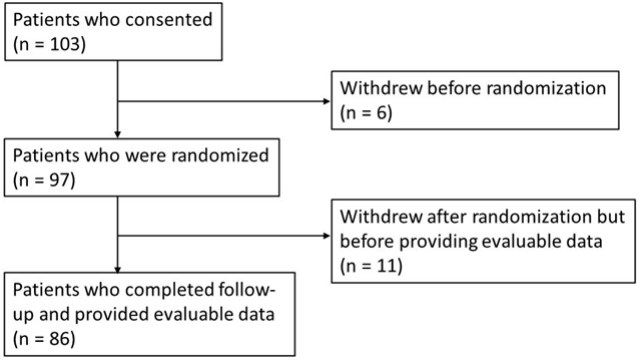
CONSORT diagram of patients who provided consent and were followed-up during the trial. Evaluable data are defined as providing at least one primary or secondary outcome measure.

A total of 103 patients were enrolled and provided consent (Figure 1). Of these, 6 patients withdrew from the study before randomization; 97 participants were randomized. Of those randomized, 11 (4 randomized to the Dashboard arm and 7 to the TAU arm) withdrew before providing evaluable data with at least one primary or secondary outcome measure. Thus, a total of 86 patients had evaluable data and were included in the primary analysis.

Table 1 shows a breakdown of the demographic characteristics of the sample who were randomized by treatment arm. A majority of the sample were women (79.4%), White (73.2%), and young with an age breakdown of <18 years (41.2%), between 18 and 25 years (36.1%), and >26 years (22.7%). Most patients had a depressive disorder (73.2%), while relatively fewer had a bipolar disorder (11.3%) or a primary anxiety disorder (15.5%). Importantly, we found no significant differences between the two treatment arms in any of these characteristics, or in the number who withdrew before providing evaluable data (all comparisons: *P*>.05). In addition, recalling the pragmatic study design in which the number of follow-up visits for each patient was determined solely based on clinical indication, there was not a significant difference in the mean number of follow-up visits between the two treatment arms (dashboard vs TAU: 8.29 vs 9.63; *t*_84_=-1.22; *P*=.22).

**Table 1.** Characteristics of patients randomized by treatment arm.^a^

	Dashboard arm (n=49)	TAU^b^ arm (n=48)	Total (n=97)
Sex, n (%)			
Male	11 (22.5)	6 (12.5)	17 (17.5)
Female	38 (77.5)	39 (81.3)	77 (79.4)
Other	0 (0)	3 (6.2)	3 (3.1)
Race, n (%)			
White	34 (69.4)	37 (77.1)	71 (73.2)
Black	7 (14.3)	5 (10.4)	12 (12.4)
Asian	3 (6.1)	2 (4.2)	5 (5.1)
Other	5 (10.2)	4 (8.3)	9 (9.3)
Age, years, n (%)			
<18	21 (42.9)	19 (39.6)	40 (41.2)
18‐25	18 (36.7)	17 (35.4)	35 (36.1)
>26	10 (20.4)	12 (25.0)	22 (22.7)
Diagnosis, n (%)^c^			
Depression	35 (71.4)	36 (75.0)	71 (73.2)
Anxiety	10 (20.4)	5 (10.4)	15 (15.5)
Bipolar Disorder	4 (8.2)	7 (14.6)	11 (11.3)
Withdrew	4 (8.2)	7 (14.6)	11 (11.3)

a All differences on the patient characteristics were not significantly different between the dashboard and TAU arms (*P*>.05).

b TAU: treatment-as-usual.

c 66 of the patients with depression and 8 of the patients with bipolar disorder also had a history of comorbid anxiety.

**Figure 2.**
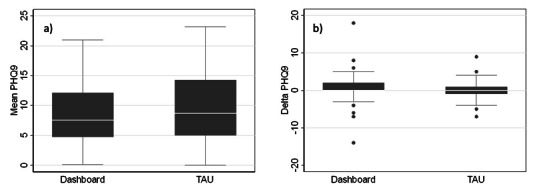
Box plots of (a) mean PHQ-9 scores over follow-up and (B) change in the PHQ-9 scores between baseline and the last available measure for patients in the dashboard versus the treatment-as-usual (TAU) arms. PHQ-9: Patient Health Questionnaire–9.

In the primary analysis, the mean PHQ-9 scores were 8.67 in the dashboard arm versus 9.56 in the TAU arm (*t*_75_=−0.72; *P*=.48; Figure 2A). Using a mixed effect linear regression model with repeated observations of the outcome (Table 2), the difference between the two arms was not statistically significant after controlling for follow-up time and patient covariates (β_Difference_=−.83; SE=1.31; *P*=.53). We further examined changes in PHQ-9 scores between baseline and the last observation (Figure 2B) and noted that the scores declined by a mean of 0.58 points for the dashboard arm versus 0.35 for the TAU arm (*t*_60_=0.20; *P*=.85). Again, using a mixed effect linear regression model this time including an interaction term between treatment arm and time, we found that the change over time was not significantly different between the two arms after controlling for the other patient covariates (β_Interaction_=.007, SE=0.01, *P*=.48). We carried out a similar set of analyses with the secondary outcomes (SF36-MHC and GAD-7 scores) and likewise did not find any significant differences between the two treatment arms (Multimedia Appendix 1).

**Table 2.** Model results comparing the dashboard versus treatment-as-usual arms considering the primary outcome and hypothesized mediators.

Model^a^	Outcome	Covariates	β (SE)	95% CI (*P* value)	*P* value
1	PHQ-9^b^ score	TrxArm^c^	−0.83 (1.21)	−3.19 to 1.53	0.49
		Time	−0.003 (0.006)	−0.015 to 0.009	0.62
2	PHQ-9 score	TrxArm	−1.14 (1.39)	−3.87 to 1.59	0.41
		Time	−0.006 (0.007)	−0.021 to 0.008	0.40
		TrxArm^a^ and Time	0.007 (0.012)	−0.017 to 0.031	0.58
3	M-CICAS^d^	TrxArm	1.00 (0.38)	0.24 to 1.76	0.01
4	WAI-SR^e^	TrxArm	−1.35 (1.88)	−5.11 to 2.41	0.48

a Models 1 and 2 are from mixed effects linear regression of the outcome as a function of the covariates shown plus age, sex, race, and diagnosis, and including a random effect for individual to account for repeated measures of the outcome. Models 3 and 4 are from linear regression models of the outcome shown as a function of the covariates shown plus patient characteristics including age, sex, race, diagnosis and length of follow-up in days.

b PHQ-9: Patient Health Questionnaire–9.

c TrxArm: treatment arm. This refers to the generic variable that can take on the values of either "dashboard" or "treatment-as-usual (TAU)", and is used to represent the variable in the model that captures the comparison of results between the two levels of this variable: the Dashboard arm vs the TAU arm.

d M-CICAS: McLean Collateral Information and Clinical Actionability Scale

e WAI-SR: Working Alliance Inventory – Short Revised.

**Figure 3.**
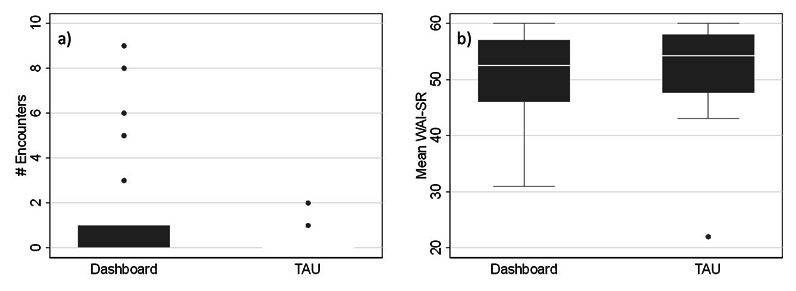
Box plots of (A) the number of encounters in which digital social activity was discussed and (B) the mean WAI-SR scores over the course of follow-up for patients in the dashboard versus treatment-as-usual (TAU) arms. WAI-SR: 12-item Working Alliance Inventory–Short Revised.

In secondary analyses, we examined whether the dashboard intervention was associated with factors that were hypothesized a priori to mediate the potential therapeutic effect of the intervention [7]. Using data from the M-CICAS to test whether the dashboard intervention was associated with an increase in the number of times that patients’ digital social activity was discussed in clinical sessions (Figure 3A), we found the mean was significantly greater in the dashboard arm versus the TAU arm (dashboard vs TAU arm: 1.07 vs 0.18; *t*_77_=2.40; *P*=.02). This difference remained significant even after controlling for the length of follow-up and patient covariates (β_Difference_=1.00, SE=0.38, *P*=.01). However, we did not find that the increased discussion of the patients’ digital social activity led to any increase in the number of encounters in which a clinical adjustment was made as also measured using the M-CICAS, before or after controlling for the length of follow-up and patient covariates (β_Difference_=−.41, SE=0.56, *P*=.46). We then used data from the WAI-SR to examine whether the dashboard intervention was associated with differences in patient-rated therapeutic alliance compared with TAU (Figure 3B). We found there was not a significant difference in the mean patient-rated alliance scores over time between the dashboard arm and the TAU arm (dashboard vs TAU: 51.16 vs 52.18, *t*_71_=-0.59, *P*=.56), even after controlling for the length of follow-up and patient covariates (β_Difference_=−1.35, SE=1.88, *P*=.48).

In other exploratory secondary analyses, we re-analyzed the data on the primary outcome (PHQ-9) after stratifying by age at baseline at ages <18, 18‐25, and >25 years, corresponding with typical developmental milestones through adolescence and young adulthood. We did not find any significant differences in PHQ-9 scores over the course of follow-up between the two treatment arms for any of the age sub-groups, although the resulting sample sizes in each group were relatively small (results not shown). In addition, we re-analyzed the primary outcome data after stratifying for an indicator of clinician engagement with the electronic dashboard during the trial. Here, we re-analyzed the data specifically within the sub-group of patients (n=24 vs 23 in the dashboard and TAU arms, respectively) cared for by clinicians who reported on the M-CICAS using the electronic dashboard at more than one encounter. Again, we did not find any significant differences in PHQ-9 scores over the course of follow-up between the two treatment arms in this sub-group (results not shown).  

## Discussion

This randomized trial tested whether providing clinicians with an electronic dashboard that displayed information about their patients’ digital activity on social media and electronic communications platforms would facilitate more informed and collaborative treatment decisions and improve outcomes for individuals with mood or anxiety disorders. We did not find evidence that providing such a dashboard significantly improved outcomes. However, it tended to promote greater discussion about the patients’ digital social activity during the course of treatment, and it did not negatively impact patients’ therapeutic alliance with their clinicians. The latter is noteworthy because it was unclear at the onset of the trial whether providing the dashboard would facilitate dialogue and greater alliance between patients and their clinicians or, alternatively, harm alliance by raising concerns among patients about their privacy.

There is growing interest in the role of social media in influencing mental health, especially among adolescents and young adults [40]. However, there have been relatively few efforts to evaluate the potential impact of systematically tracking patients’ digital social activity and using this information in clinical care. One study reported on the development of an electronic dashboard for monitoring digital activity of patients on a variety of social media platforms but did not test it in clinical care [27,28]. Another study asked patients to share at least one type of social media (eg, Facebook wall posts) or digital data (eg, Google or YouTube video search queries, or smartphone metrics such as steps walked or screen status) before clinic visits. They tested whether providing a dashboard that displayed the shared information to clinicians improved health-related quality of life or depressive and anxiety symptoms. Although it was feasible to use the dashboard in routine clinical care, it did not improve patient outcomes [29]. We adapted a commercially available mobile app that allowed us to comprehensively monitor patients’ self-selected social media activity and electronic communications platforms to provide detailed information on what platforms they were using, when they were using them, as well as on specific content posted on these platforms. Still, we observed broadly similar findings as the previous study.

Using the app-based approach to monitor patients’ digital social activity presented certain challenges due to concerns about potential intrusion into patients’ privacy. We adopted a number of strategies to minimize these concerns. We developed a pipeline for collecting and processing the social activity data that prioritized protecting the patients’ privacy by removing personal identifiers from the data streams and linking the data to an anonymous study ID, processing and presenting the data behind the Johns Hopkins firewall, and looking at the processed data only when clinicians were in session with patients. In addition, we allowed patients to decide which social media platforms to share through the app, the clinicians to decide when to use the dashboard in clinical sessions, and the clinicians and patients to decide together when to review specific content. These decisions promoted patient control over what information was revealed, with the goal of facilitating trust and greater collaboration between clinicians and patients in using the dashboard to inform treatment sessions. In the end, these strategies were helpful in building trust with patients, but recruitment remained challenging and prolonged due to lingering questions about privacy. However, we observed that, for patients who agreed to participate, these concerns were not apparent and did not impact the alliance they had with their clinicians.

The COVID-19 pandemic also had a significant impact on the start of the trial and on-going recruitment. We were not able to randomize our first patient until a half-year into the pandemic, and we had to modify procedures to accommodate the evolving clinical landscape, which included the rapid transition to telehealth. Due to this transition, we had to recruit and follow patients virtually, which led to challenges that slowed recruitment and made it more difficult to follow-up patients over time, contributing to the losses in follow-up.

There were several other limitations of the study that merit consideration. Our decision to allow patients to control what information they shared and clinicians to decide when to use the dashboard in clinical sessions, while good intentioned, may have diminished the ability of the intervention to have a meaningful clinical impact. It is possible that clinically salient information from the patients’ digital social activity was not shared or discussed, and this could have reduced any potential benefit of the intervention. We did not have a measure of how often the clinicians and patients used the dashboard and reviewed its contents during clinical sessions, and as a result, we could not tell how engaged they were with the intervention. Instead, we had a measure of how often digital social activity was discussed in these clinical sessions. While we did show a difference with the dashboard, the number of times that such discussions came up in clinical sessions overall was relatively modest. Future studies of this approach should consider strategies to incentivize patients to share their digital social activity with the dashboard and clinicians to use the dashboard as part of every clinical session, much like they are encouraged to use other routine measures in traditional measurement-based care.

Another limitation is that patients were recruited into the study at any stage of their illness, which may have reduced the ability to detect a clinical benefit of the dashboard intervention. Moreover, our primary outcome measure using the PHQ-9 may not have been optimal for detecting such a benefit. While the PHQ-9 is widely used in studies of depression and anxiety, there are likely multiple complex factors that contribute to its trajectories over time, limiting the ability of the dashboard intervention from having a meaningful impact over only 3 months of follow-up. Future studies may want to consider other wellness or other non-traditional outcome metrics to measure the impact of the intervention, as well as longer follow-up durations to allow sufficient time for the intervention to have a meaningful impact. Despite the challenges and equivocal findings with regard to the primary outcomes, we believe the trial provided valuable lessons that motivate further research into this important area. We found that it was feasible to implement the novel dashboard intervention in real-world clinical care, it did not negatively impact patient-clinician alliance despite concerns over privacy, and using the intervention did increase discussions about the patients’ digital social activity that may have impacted their mental health as hypothesized. As a result, we contend more research is warranted to improve the clinical utility of the electronic dashboard and further test the intervention in real-world clinical care.

## Supplementary material

10.2196/74212 Multimedia Appendix 1 Box plots of the (a) mean SF36-MCS scores over follow-up and (b) change in SF36-MCS scores between baseline and last available measure for patients in the dashboard versus treatment-as-usual (TAU) arms.

## Abbreviations

GAD-7: 7-item General Anxiety Disorder-7
LIWC: linguistic inquiry and word count
M-CICAS: McLean Collateral Information and Clinical Actionability Scale
PHQ-9: 9-item Patient Health Questionnaire-9
SF36-MHC: 13-item Short-Form 36 Health Survey Mental Health Component
TAU: treatment-as-usual
WAI-SR: 12-item Working Alliance Inventory–Short Revised
