# Supplementary figures and images for: Dashboard Intervention for Tracking Digital Social Media Activity in the Clinical Care of Individuals With Mood and Anxiety Disorders: Randomized Trial
Source: JMIR Ment Health. 2025 Nov 11;12:e74212. doi: 10.2196/74212 (PMC12604431; doi:10.2196/74212)

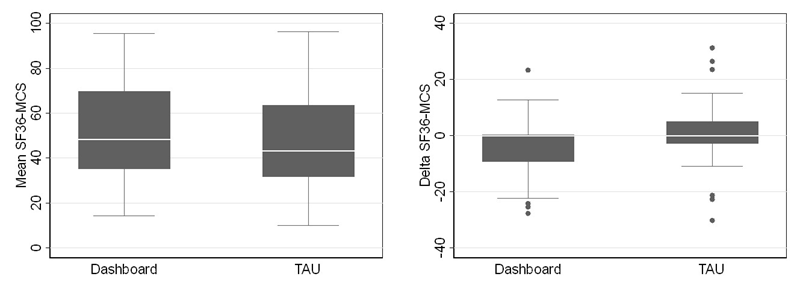

Supplement: Multimedia Appendix 1 [file mental-v12-e74212-s001.png]
